# Identifying optimal reference genes for real-time quantitative polymerase chain reaction in human myocardial tissues

**DOI:** 10.1093/cvr/cvae194

**Published:** 2024-09-06

**Authors:** Maria Camacho-Encina, Laura K Booth, Rachael Redgrave, Minna Honkanen-Scott, William E Scott, Carmen Martin-Ruiz, Guy MacGowan, Sarah Richardson, John Dark, Simon Tual-Chalot, Gavin D Richardson

**Affiliations:** Vascular Medicine and Biology Medicine Theme, Biosciences Institute, Newcastle University, Newcastle upon Tyne NE1 3BZ, UK; Vascular Medicine and Biology Medicine Theme, Translational and Clinical Research Institute, Newcastle University, Newcastle upon Tyne NE1 3BZ, UK; Vascular Medicine and Biology Medicine Theme, Biosciences Institute, Newcastle University, Newcastle upon Tyne NE1 3BZ, UK; Regenerative Medicine, Stem Cells, and Transplantation Theme, Translational and Clinical Research Institute, Newcastle University, Newcastle upon Tyne NE1 3BZ, UK; Regenerative Medicine, Stem Cells, and Transplantation Theme, Translational and Clinical Research Institute, Newcastle University, Newcastle upon Tyne NE1 3BZ, UK; Vascular Medicine and Biology Medicine Theme, Biosciences Institute, Newcastle University, Newcastle upon Tyne NE1 3BZ, UK; Vascular Medicine and Biology Medicine Theme, Biosciences Institute, Newcastle University, Newcastle upon Tyne NE1 3BZ, UK; Islet Biology Exeter (IBEx), Exeter Centre of Excellence for Diabetes Research (EXCEED), Department of Clinical and Biomedical Sciences, University of Exeter Medical School, Exeter EX1 2LU, UK; Vascular Medicine and Biology Medicine Theme, Biosciences Institute, Newcastle University, Newcastle upon Tyne NE1 3BZ, UK; Vascular Medicine and Biology Medicine Theme, Biosciences Institute, Newcastle University, Newcastle upon Tyne NE1 3BZ, UK; Vascular Medicine and Biology Medicine Theme, Biosciences Institute, Newcastle University, Newcastle upon Tyne NE1 3BZ, UK

Myocardial senescence and the associated increase in inflammation via the senescence-associated secretory phenotype (SASP) have been demonstrated to contribute significantly to the pathogenesis and pathophysiology of various cardiovascular diseases (CVDs). In preclinical models, myocardial senescence accumulates with age and following acute cardiac stress, and elimination of senescence is associated with improved cardiac function and reduced disease pathophysiology.^1–4^ As such, there is a growing interest in targeting myocardial senescence as a potential therapeutic approach for CVDs clinically, or alternatively assessing tissue senescence or circulating SASP as prognostic biomarkers for cardiac care.^2,5^

While microarray and RNA-seq technologies enable high-throughput genome-wide transcriptome analysis, real-time quantitative polymerase chain reaction (RT-qPCR) is preferred for small-scale gene expression studies due to its exceptional sensitivity, wide dynamic range, speed, and high reproducibility. However, RT-qPCR validity depends on selecting appropriate reference genes.^6^ Recent systematic analyses indicate that only about 15% of published studies provide evidence that their chosen reference genes are stably expressed.^7^ This lack of validation can significantly affect the quality and validity of data, particularly in studies aimed at identifying biomarkers, diagnostic tools, and therapeutic targets.^8^ Therefore, it is crucial for such studies to carefully select appropriate reference genes that remain unaffected by the biological differences within the study cohort. The ongoing reproducibility crisis underscores the critical role of reference gene choice in ensuring the reliability of molecular research outcomes. The selection of appropriate reference genes may be particularly challenging in the context of myocardial remodelling, senescence, and aging as myocardial remodelling and senescence are associated with changes in fundamental processes including respiration, protein synthesis, cytoskeletal organization, and ribosomal RNA expression.^9^

To the best of our knowledge, a set of appropriate reference control genes that are expressed stably within a cohort of human cardiac samples representing a high degree of heterogeneity in senescence and CVD has yet to be characterized. This study therefore aimed to identify optimal reference genes for RT-qPCR normalization in human myocardial tissues across different states of senescence and disease.

Samples of comparable regions of left ventricular (LV) myocardium were obtained from 16 clinically declined donor hearts under Quality in Organ Donation Tissue Bank (QUOD) research ethics (23/NW/0097). All donors or donor families had appropriate research consent obtained, and this investigation conforms to the principles outlined in the Declaration of Helsinki. This cohort, heterogeneous in demographics and clinical history (*Figure 1A*), allowed the robust identification of reference genes for which the expression is unaffected by characteristics that are typically variable when using human samples. Based on donor clinical history, myocardial samples represented a span of CVDs including hypertension (donors D, F, N, and O), evidence of LV hypertrophy (donors D and N), sinus bradycardia (donors D and L), angina (donor L), and coronary artery disease (donor N). Furthermore, donors E, F, G, K, L, N, and P died following a cardiac arrest (*Figure 1A*). All other donors had no recorded history of CVD. As clinical data can be incomplete and individuals could be suffering from an undiagnosed CVD, we next assessed tissue variability using immunohistochemistry for remodelling markers pro-B-type natriuretic peptide (proBNP) and collagen deposition, and myocardial senescence using expression of the classical senescence markers p16^Inl4a^ and p21^Cip1^ (*Figure 1B*). As would be expected based on the clinical data, these studies revealed extensive heterogeneity in the expression of markers associated with myocardial remodelling. Additionally, large variations were observed in the expression of senescence markers p16^Ink4a^ and p21^Cip1^, as would be expected given the association between senescence, disease, and age. Importantly, these data underscore the suitability of this cohort for identifying stable reference gene expression in a heterogenous cohort (*Figure 1C*).

**Figure 1:**
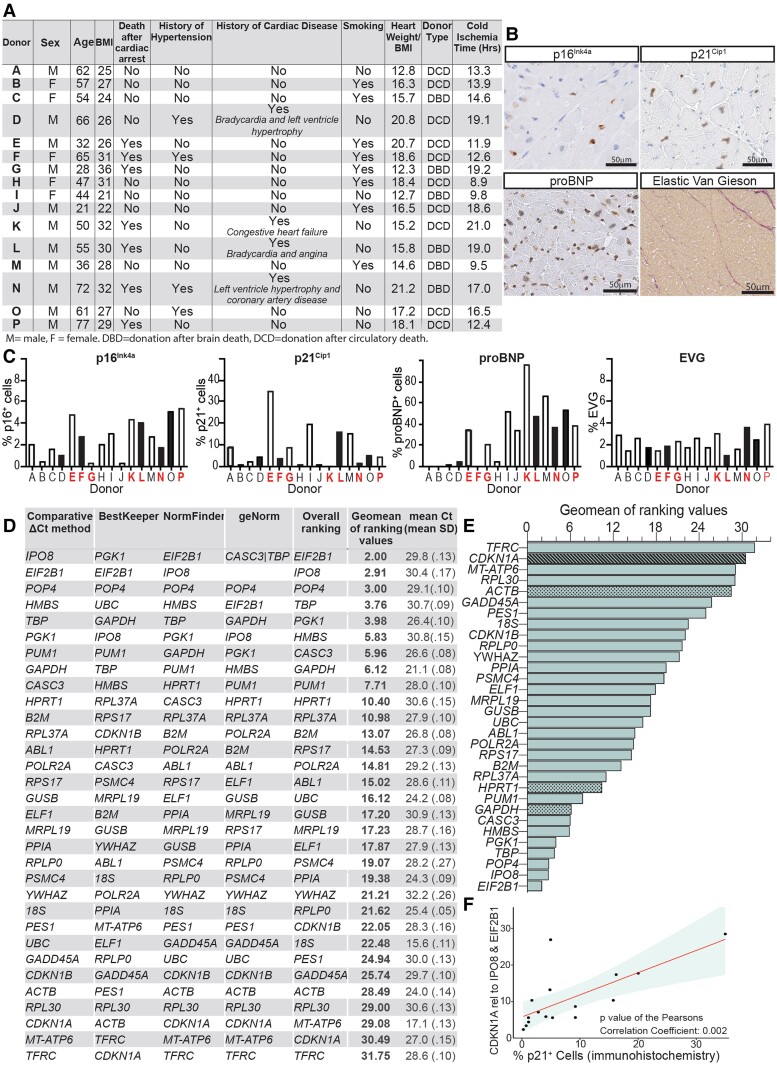
(*A*) Characteristics of the donors included in this study. (*B*) Representative images of immunohistological analysis of p16^Ink4a^ (clone E6H4, Ventana), p21^Cip1^ (clone 12D1, Cell Signalling) and proBNP (clone C11, Abcam ab239519), and staining with Elastin van Gieson (Atom Scientific, Miller EVG staining kit) in donor LV myocardium. (*C*) Quantification of the percentage of cells expressing each protein for each of the 16 donor samples (A–P), demonstrating a range of expression. Black bars indicate history of CVD or hypertension, and red letters indicate donor died after a cardiac arrest. (*D*) Table showing ranking order from RefFinder and the average RT-qPCR Ct values for all 32 candidate reference genes, with each individual gene Ct value based on data from 3 technical replicates. (*E*) Comprehensive ranking of the 32 reference genes included in the TaqMan Array panel using RefFinder web tool, calculated according to the geometric mean of the genes’ weighting. The least stable genes are on the top, and the most stable genes are on the bottom. Dot-patterned bars represent common reference genes *GAPDH*, *HPRT1*, and *ACTB* while the stripe-patterned bar represents *CDKN1A* gene, a controller of senescence. (*F*) *CDKN1A* expression significantly correlates with the percentage of cells expressing p21^Cip1^ using *IPO8* and *EIF2B1* as reference genes for normalization.

We then evaluated reference gene expression and stability in adjacently located LV samples through the Human Endogenous Control TaqMan® Array (Thermo Fisher Scientific, cat. no. 4396840), which comprises 32 potential reference gene candidates. The median Ct (threshold cycle) for each candidate reference gene from each donor was used to analyse relative expression stability using the comparative ΔCt method, geNorm, NormFinder, and BestKeeper algorithms, all available within RefFinder web tool^10^ (*Figure 1D* and *E*). The comparative ΔCt method assesses stability using the mean of SD values derived from a comparison between a particular reference gene and any other candidate. Based on this, the top five most stable genes were *IPO8* > *EIF2B1* > *POP4* > *HMBS* > *TBP*. BestKeeper uses pairwise correlations of the expression levels of all candidate reference genes and combines the highly correlated candidate into an index. The software calculates the SD, percentage covariance, and power of the candidates to help determine the most stable gene. The five most stable genes provided by BestKeeper were *PGK1* > *EIF2B1* > *POP4* > *UBC* > *GAPDH*. geNorm finds the most stable reference gene by comparison of the gene stability measure (*M*), which is calculated as the average pairwise variation of each gene in relation to all other reference genes in the analysis. At each step, the gene with the highest *M* value is excluded, and *M* values are recalculated for the remaining genes, resulting in a ranking of the most stable genes (i.e. lower *M* values reflect higher stability). This process is followed in a stepwise manner until the two most stable genes, which cannot further be ranked, remain. According to geNorm, the top five were *CASC3* | *TBP* > *POP4* > *EIF2B1* > *GAPDH*. Unlike geNorm, NormFinder provides a direct measure for the estimated expression variation (stability), which allows for the evaluation of the systematic error introduced when using the gene. The algorithm uses a model-based strategy to estimate the overall variation of both the reference genes and between subgroups of samples, and then ranks them in order. NormFinder selected *EIF2B1* > *IPO8* > *POP4* > *HMBS* > *TBP* as the five most stable genes. Based on all four programmes, RefFinder provides a comprehensive ranking by assigning weightings to each reference gene and calculating the geometric mean of the weightings, with a lower geometric mean representing higher stability of the candidate gene. Using this approach, the top five most stable genes were *EIF2B1*, *IPO8*, *POP4*, *TBP*, and *PGK1* (*Figure 1D* and *E*). Highlighting the utility of the current study, commonly used reference genes *GAPDH*, *HPRT1*, and *ACTB* were identified as relatively unstable between donors, ranking 8th, 10th, and 28th, respectively, in terms of their stability (*Figure 1D*, dotted bars).

When selecting a panel of reference control genes, it is crucial to ensure that these genes are not transcriptionally coregulated, as coregulated genes may respond similarly to stimuli, which can lead to falsely identifying genes as stable when employing methods that rely on pairwise comparisons.^11^ Using STRING to analyse the 10 most stable genes, we identified PGK1 (5th position) and GAPDH (8th position) had a confidence interaction score of ≥0.7, suggesting potential coregulation. Hence, it is advisable not to employ these genes in combination as reference controls. As the control array included *CDKN1A*, the gene encoding p21^Cip1^ (*Figure 1E*, stripe-patterned bar), we used this to validate the two most stable genes. When using both *IPO8* and *EIF2B1* together as the reference, the relative expression of *CDKN1A* transcript significantly and positively correlated with p21^Cip1^ protein expression [*P* value of the Pearson correlation coefficient (*r*) = 0.002] within our sample cohort (*Figure 1F*), indicating these two genes provide a reliable normalizer of gene expression for studies of human LV, when divergent states of senescence and characteristics of disease are present. Given our data showing the instability of *ACTB* expression in LV obtained from individual donors, together with observations that *ACTB* expression is increased with age in non-cardiac tissues,^12^ we next identified if the variation in *ACTB* expression is related to differences in myocardial senescence within the samples. A significant positive correlation (*P* = 0.03) was observed between *ACTB* expression and p21^Cip1^ protein.

In summary, for accurate, robust, and reproducible analysis of differential gene expression in human myocardium, the data from this study strongly recommend using both *EIF2B1* and *IPO8* as reference controls. These genes have demonstrated stability across various clinical and biological parameters. By utilizing *EIF2B1* and *IPO8* as reference genes, researchers can enhance the reliability and validity of their gene expression analyses in studies of human myocardial biology.

## Authors’ contributions

M.C.-E. led the study. M.C.-E., L.K.B., and R.R. performed the experiments. M.H.-S. and W.E.S. oversaw the donor heart collection and processing within QUOD. G.D.R., M.C.-E., S.T.-C., C.M.-R., G.M., S.R., and J.D. contributed to the study design and funding. M.C.-E. and G.D.R. designed and supervised the study. All contributed to the analysis and the writing of the manuscript.

## Data Availability

The data underlying this article are available in the article.
